# Mid-term (up to 12 years) clinical and echocardiographic outcomes of percutaneous transvenous mitral commissurotomy in patients with rheumatic mitral stenosis

**DOI:** 10.1186/s12872-021-02175-3

**Published:** 2021-07-28

**Authors:** Yahya Dadjo, Maryam Moshkani Farahani, Reza Nowshad, Mohsen Sadeghi Ghahrodi, Alireza Moaref, Javad Kojuri

**Affiliations:** 1 Cardiology Department, Clinical Research Center, Namazi Hospital, Shiraz University of Medical Sciences, Zand St., Cardiology Office, Shiraz, Iran; 2 Atherosclerosis Research Center, Baqiyatallah University of Medical Sciences, Tehran, Iran; 3 Cardiovascular Research Center, Shiraz University of Medical Sciences, Shiraz, Iran

**Keywords:** Mitral valve stenosis, Percutaneous transvenous mitral commissurotomy, Mid-term outcome, Adverse cardiac events, Restenosis

## Abstract

**Background:**

Rheumatic heart disease (RHD) is still a concerning issue in developing countries. Among delayed RHD presentations, rheumatic mitral valve stenosis (MS) remains a prevalent finding. Percutaneous transvenous mitral commissurotomy (PTMC) is the intervention of choice for severe mitral stenosis (MS). We aimed to assess the mid-term outcome of PTMC in patients with immediate success.

**Methods:**

In this retrospective cohort study, out of 220 patients who had undergone successful PTMC between 2006 and 2018, the clinical course of 186 patients could be successfully followed. Cardiac-related death, undergoing a second PTMC or mitral valve replacement (MVR) were considered adverse cardiac events for the purpose of this study. In order to find significant factors related to adverse cardiac outcomes, peri-procedural data for the studied patients were collected.The patients were also contacted to find out their current clinical status and whether they had continued secondary antibiotic prophylaxis regimen or not. Those who had not suffered from the adverse cardiac events were additionally asked to undergo echocardiographic imaging, in order to assess the prevalence of mitral valve restenosis, defined as mitral valve area (MVA) < 1.5 cm^2^ and loss of ≥ 50% of initial area gain.

**Results:**

During the mean follow-up time of 5.69 ± 3.24 years, 31 patients (16.6% of patients) had suffered from adverse cardiac events. Atrial fibrillation rhythm (*p* = 0.003, HR = 3.659), Wilkins echocardiographic score > 8 (*p* = 0.028, HR = 2.320) and higher pre-procedural systolic pulmonary arterial pressure (*p* = 0.021, HR = 1.031) were three independent predictors of adverse events and immediate post-PTMC mitral valve area (IMVA) ≥ 2 cm^2^ (*p* < 0.001, HR = 0.06) was the significant predictor of event-free outcome. Additionally, follow-up echocardiographic imaging detected mitral restenosis in 44 patients (23.6% of all patients). The only statistically significant protective factor against restenosis was again IMVA ≥ 2 cm^2^ (*p* = 0.001, OR = 0.240).

**Conclusion:**

The mid-term results of PTMC are multifactorial and may be influenced by heterogeneous peri-procedural determinants. IMVA had a great impact on the long-term success of this procedure. Continuing secondary antibiotic prophylaxis was not a protective factor against adverse cardiac events in this study. (clinicaltrial.gov registration: NCT04112108).

**Supplementary Information:**

The online version contains supplementary material available at 10.1186/s12872-021-02175-3.

## Introduction

Rheumatic heart disease (RHD), despite the decrease in its prevalence in some parts of the world, continues to be a major medical concern in developing countries [1, 2]. Among delayed RHD presentations, rheumatic mitral valve stenosis remains a common finding. It accounts for the great majority of all cases of mitral stenosis (MS) even in developed nations [3, 4]. Since the early 1980s with the introduction of the Inoue balloon, the management of MS has changed considerably, and percutaneous transvenous mitral commissurotomy (PTMC) has become the mainstay of treatment for symptomatic patients with severe MS and favorable valve anatomy [5, 6].

Although the immediate success rate of PTMC is relatively the same as that of surgical commissurotomy techniques, the long-term results and appropriate patient selection for this procedure remain debatable [7, 8]. Regarding morphological features, several echocardiographic criteria have been developed, with the semi-quantitative Wilkins score being the most widely used [9, 10]. Nonetheless, several randomized trials have shown that other characteristics may be just as important in predicting the incidence of adverse clinical events in the long-term [11–18]. But because of differences among trials in study design, the criteria and techniques used, and the duration of follow-up, the success rate and the reported predictive factors have not been always similar. The same issues surround the study of mitral valve restenosis, which is expected at some point in the course of follow-up due to the persistence of underlying rheumatic pathology. But again, the timing of appearance and incidence rates have been shown to be multifactorial [19, 20].

Because only one valid study, to the best of our knowledge, is available in our country (Iran) on the late results of PTMC [16], the present study was designed to determine the mid-term outcome of PTMC at local tertiary centers, and to identify which characteristics are potentially helpful in predicting adverse cardiac events as well as mitral valve restenosis. We also investigated whether a continuing secondary antibiotic prophylaxis regimen had any positive effects on the long-term outcome in these patients.

## Material and methods

### Study population and design

This research was designed as single-technique (Inoue balloon), multicenter retrospective cohort study. The target group of this study consisted of 220 patients with symptomatic rheumatic MS who had undergone PTMC at 4 hospitals with similar procedural setups and registry systems (Baqiyatallah, Namazi, Shahid-Faghihi and Kowsar) under the supervision of Baqiyatallah and Shiraz Universities of Medical Sciences between April 2006 and January 2018.

The inclusion criteria for the participants were: (1) age more than 20 years at the time of PTMC; (2) immediate post-PTMC mitral valve area (IMVA), obtained through the first session of echocardiography during the first day after PTMC, ≥ 1.5 cm^2^, or for lower values, at least 50% increase in pre-PTMC mitral valve area (MVA); 3) an initial cardiovascular event-free period of at least 6 months after the procedure.

The exclusion criteria consisted of: (1) more than 2 + mitral regurgitation (MR) immediately after the procedure; (2) immediate cardiovascular event during the hospital stay after the procedure; (3) more than mild aortic stenosis or sufficiency before PTMC; (4) history of previous PTMC or surgical mitral procedures.

The study design and steps were approved by the ethics committees at both Baqiyatallah and Shiraz Universities of Medical Sciences.

### PTMC technique

All PTMC procedures were done with an Inoue balloon catheter via anterograde trans-septal approach in all patients. Right and left cardiac catheterization was performed before and during the procedure to assess hemodynamic changes. Optimal balloon size (in millimeters) was estimated with the height (cm)/10 + 10 formula. The balloon was inflated in a step-wise fashion from lower to higher volumes. After each inflation, changes in trans-mitral mean pressure gradient (TMPG) and the degree of mitral regurgitation (MR) were monitored. Based on interventionist’s judgement and in order to achieve optimal results, balloon inflation could be continued up to 1–2 mm more than the estimated size. In the last stage, left ventriculography was conducted to assess the degree of final MR.

### Echocardiography

All of the studied patients, for whom rheumatic MS had been previously by echocardiography confirmed, underwent transthoracic Doppler echocardiography (TTE) in the week before PTMC. MVA was measured by planimetry in the short axis view, and also by pressure half-time quantification when atrial fibrillation (AF) or MR were not serious enough to interfere with its interpretation. The degree of MR was reported on a semi-quantitative scale of 0 to 4 based on Doppler color flow mapping. Mitral valve morphology was also evaluated with the standard Wilkins echocardiographic scoring system. Pulmonary arterial pressure was estimated by measuring systolic pulmonary arterial pressure (sPAP) in mmHg based on the trans-tricuspid regurgitation jet. Other significant variables in this study were left atrial diameter in centimeters, and mean trans-mitral pressure gradient (TMPG) in mmHg.

Each patient also had one session of transesophageal echocardiography during the day before the procedure in order to rule out thrombi in the left atrium or the left atrial appendage. Another session of TTE was performed during the first day after the procedure to confirm the immediate success of PTMC, also to obtain immediate post-PTMC values and at last to rule out acute complications such as severe MR or cardiac tamponade.

### Follow-up

The clinical condition of the patients was recorded as their New York Heart Association (NYHA) functional class before the procedure. Their clinical status was also followed in their inpatient and also outpatient records, as there had been an annually planned clinical visit and TTE evaluation for the studied patients, in all of our centers. For the purpose of this study, the patients or their first-degree relatives were again contacted by telephone in order to record the occurrence and exact timing of any adverse cardiac events, and also to reassess their current NYHA functional class status. Adverse cardiac events considered significant for the purpose of this study included: (1) cardiac-related death, (2) another session of PTMC, or (3) surgical procedure of mitral valve replacement (MVR). The participants were also questioned whether they had continued secondary antibiotic prophylaxis regimen or not. Only standard regimens of intramuscular injection of 1.2 million units of penicillin G benzathine every 4 weeks, or in case of allergy to penicillin, usage of azithromycin (250 mg orally once daily) or erythromycin (250 mg twice daily) were considered valid in this study. Furthermore, Patients who had been event-free until that time were asked to undergo another TTE evaluation after providing their informed consent.

### Statistical analysis

Continuous variables are reported as the mean ± SD. Categorical and nominal variables are shown as the number and percentage, and were pooled in some cases in order to facilitate the interpretation of the results. Initially, univariable Cox proportional hazards regression analysis was used to assess the relationship between variables and adverse cardiac events at follow-up. In the next stage, Cox multivariable regression was also performed to single out the independent variables. To evaluate the variables for the occurrence of restenosis during follow-up, uni- and multivariable logistic regression analysis was used. In order to enter independent variables into the multivariable analyses, certain *p* value cut-offs based on univariable analysis results were implemented at each stage. Receiver operating characteristic (ROC) curves were generated to determine the best cut-off point for IMVA in interpreting the results. An additional analysis was performed with the help of paired sample t-test and Wilcoxon signed-rank test to evaluate the differences in the characteristics of the Event-free patients between the beginning point of the study and the time of last echocardiographic and clinical follow-up. For all analyses a *p* value of 0.05 or less was considered statistically significant. All data analyses were done with IBM SPSS Statistics version 23 software.

### Patient and public involvement statement

This research was based on active registry of patients with mitral stenosis, all patients were asked to freely reports their comments and problems during follow up and all tests specifically needed for research including visits, laboratory and echocardiographic studies were done free of charge for all patients.

Patients were completely informed about aims, scopes and methods of research before and during conduction, but they had no active role in design or dissemination of results.

The results of all evaluations were reported to patients and they were referred to subspecialist for proper managements, whenever it was needed (Additional file 1).

## Results

It is worthy of note that aside from 220 cases included in this study, based on our database, the rate of early complications in our centers was around 6% (14 patients). The causes included occurrence of cardiac tamponade, immediate severe MR or rupture of left atrium during the procedure (prompting urgent surgical intervention) as well as PTMC failure mainly due to failed puncture of interatrial septum, failed attempt or limited success in ballooning the mitral valve, for which another session of intervention or surgery was needed in the following few weeks or months.

### Clinical follow-up

**Fig. 1:**
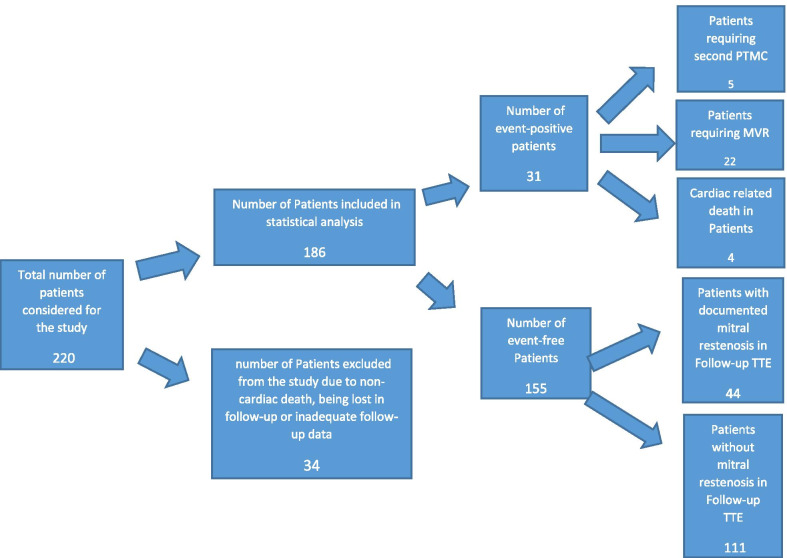
Distribution of the studied population

Out of 220 patients who met the inclusion criteria, the clinical course of 186 patients could be successfully followed. Mean follow-up time was 5.69 ± 3.24 years. Their primary characteristics are summarized in Table 1. Of the remaining patients, 12 were lost in follow-up: 3 had died of non-cardiac causes, and for 19 patients the required follow-up data could not be obtained in full (Fig. 1).

**Table 1:** Primary characteristics of the patients

Characteristics	All patients	Event-free group	Event-positive group	*p* value
Number	186	155	31	
*Demographic*				
Age (in years)	46.97 ± 11.67	46.43 ± 11.54	49.65 ± 12.15	0.08
Female sex	152 (81.7%)	125 (80.6%)	27 (87%)	0.19
*Clinical*				
NYHA functional class > 2	142 (76.3%)	120 (77.4%)	22 (70.9%)	0.57
AF cardiac rhythm	48 (25.8%)	32 (20.6%)	16 (51.6%)	< 0.001
Antibiotic (penicillin) prophylaxis	61 (32.7%)	55 (35.4%)	6 (19.3%)	0.03
*Echocardiographic (Pre-PTMC)*				
E.F (%)	55.60 ± 4.45	55.69 ± 4.21	55.16 ± 5.55	0.11
Left atrial diameter(cm)	4.48 ± 0.58	4.46 ± 0.61	4.56 ± 0.43	0.10
TMPG (mmHg)	15.43 ± 6.86	15.28 ± 6.76	16.21 ± 7.4	0.64
MVA (cm2)	1.03 ± 0.20	1.02 ± 0.20	0.99 ± 0.21	0.55
MR ≥ degree 1	112 (60.2%)	91 (58.7%)	21 (67.7%)	0.38
Wilkins score	8.12 ± 1.21	8.02 ± 1.20	8.61 ± 1.14	0.021
sPAP (mmHg)	43.69 ± 12.9	42.38 ± 12.45	50.33 ± 13.28	0.003
*Echocardiographic (Post-PTMC)*				
IMVA (cm2)	2.03 ± 0.37	2.08 ± 0.35	1.76 ± 0.35	< 0.001
IMR: degree 2	35 (18.8%)	23 (14.8%)	12 (38.7%)	0.002
ITMPG (mmHg)	5.49 ± 1.59	5.43 ± 1.56	5.75 ± 1.73	0.45

Values are mean ± SD or number (percentage in total). *NYHA* New York Heart Association, *AF* atrial fibrillation, *EF* ejection fraction, *TMPG* mean trans-mitral pressure gradient, *MVA* mitral valve area, *MR* mitral regurgitation, *sPAP* systolic pulmonary arterial pressure, *IMVA* immediate post-PTMC mitral valve area, *IMR* immediate post-PTMC mitral regurgitation, *ITMPG* immediate post-PTMC mean trans-mitral pressure gradient

Out of a total of 186 patients, 31 (16.6%) suffered from one of adverse cardiac events during the follow-up period. The frequency of each cardiac event was: second PTMC session, 5 (2.6%); MVR, 22 (11.8%); and cardiac-related death, 4 (2.1%). The mean interval between PTMC and events was 4.67 ± 2.43 years. The remaining 155 patients (83.3%) were considered the event-free. (Table 1).

In the next step, demographic, clinical and echocardiographic characteristics were compared between the event-positive and event-free groups. Univariable analysis was done with a univariable Cox proportional hazards model. This analysis showed that AF cardiac rhythm (*p* < 0.001), lower IMVA (*p* < 0.001), higher degree of immediate MR (*p* = 0.002), Wilkins score > 8 (*p* = 0.021), higher baseline sPAP (*p* = 0.003), and lack of continuing secondary penicillin prophylaxis (*p* = 0.03) had a statistically significant association with incidence of adverse cardiac events. The results of this analysis are shown in Table 2.

**Table 2:** Univariate Cox proportional hazards regression results for variables predictive of adverse cardiac events

Variables	Hazard ratio	95% CI	*p* Value
Age	1.026	0.997–1.057	0.08
Male Sex	0.458	0.138–1.501	0.19
NYHA functional class > 3	1.249	0.575–2.713	0.57
AF Rhythm	3.459	1.699- 7.042	< 0.001
Antibiotic (penicillin) prophylaxis	0.386	0.158–0.943	0.03
E.F	0.942	0.874–1.015	0.11
Left atrial diameter	1.582	0.906–2.764	0.10
TMPG	0.988	0.938- 1.041	0.64
MVA	0.601	0.111 -3.236	0.55
MR ≥ 1	1.397	0.658–2.968	0.38
Wilkins score > 8	2.787	1.365–5.691	0.021
sPAP	1.037	1.012–1.062	0.003
IMVA	0.071	0.024- 0.211	< 0.001
IMVA ≥ 2 cm2	0.054	0.016–0.178	< 0.001
IMR:degree 2	3.607	1.743–7.464	0.002
ITMPG	1.083	0.879–1.334	0.45

*CI* confidence interval, *NYHA* New York Heart Association, *AF* atrial fibrillation, *EF* ejection fraction, *TMPG* mean trans-mitral pressure gradient, *MVA* mitral valve area, *MR* mitral regurgitation, *sPAP* systolic pulmonary arterial pressure, *IMVA* immediate post-PTMC mitral valve area, *IMR* immediate post-PTMC mitral regurgitation, *ITMPG* immediate post-PTMC mean trans-mitral pressure gradient

Then, multivariable Cox regression analysis was applied for the variables with *p* value < 0.05 in the univariable analysis in order to identify which variables from the previous stage remained significant. This analysis disclosed that only AF rhythm (*p* = 0.003), IMVA < 2 cm^2^ (*p* < 0.001), high pre-PTMC sPAP (*p* = 0.021), and Wilkins echocardiographic score > 8 (*p* = 0.028) were the independent variables that were predictive of adverse cardiac events. The results of the multivariable analysis are summarized in Table 3.

**Table 3:** Multivariate Cox analysis results for variables predictive of adverse cardiac events

Variables	Hazard ratio	*p* Value
AF Rhythm	3.659	0.003
Antibiotic (Penicillin) prophylaxis	0.922	0.88
Wilkins score > 8	2.320	0.028
sPAP	1.031	0.021
IMVA ≥ 2 cm2	0.060	< 0.001
IMR: degree 2	1.270	0.55

*AF* atrial fibrillation, *sPAP* systolic pulmonary arterial pressure, *MVA* mitral valve area, *MR* mitral regurgitation, *IMVA* immediate post-PTMC mitral valve area, *IMR* immediate post-PTMC mitral regurgitation

### Restenosis at follow-up

In the next step, we investigated the echocardiographic findings in the event-free group of patients in order to assess mitral restenosis rate. The mean follow-up time for this group was 5.89 ± 3.36 years. Restenosis was defined as loss of more than 50% of the initial increase in MVA and simultaneously MVA < 1.5. Of 155 event-free patients, 44 (23.6% of all patients) were found to have mitral valve restenosis.. Table 4 shows the follow-up echocardiographic and clinical findings in comparison with their respective peri-procedural values.

**Table 4:** Comparison of echocardiographic and clinical follow-up data with their respective peri-procedural values in event-free patients

Variables	Peri-procedural	*p* value	Follow-up
*Echocardiographic*			
E.F (%)	55.69 ± 4.21 (P)	0.001	54.67 ± 4.015
Left atrial diameter (cm)	4.46 ± 0.61 (P)	0.514	4.42 ± 0.788
TMPG (mmHg)	5.43 ± 1.56 (IP)	< 0.001	8.63 ± 4.99
MVA (cm2)	2.08 ± 0.35 (IP)	< 0.001	1.65 ± 0.40
MR ≥ grade 2	23 (14.8%) (IP)	< 0.001	34 (21.9%)
sPAP (mmHg)	42.38 ± 12.45 (P)	< 0.001	31.76 ± 9.40
*Clinical*			
A.F Rhythm	32 (20.6%) (P)	0.096	37 (23.8%)
NYHA functional class > 2	120 (77.4%) (P)	< 0.001	28 (18%)

*EF* ejection fraction, *TMPG* mean trans-mitral mean pressure gradient, *MVA* mitral valve area, *MR* mitral regurgitation, *sPAP* systolic pulmonary arterial pressure, *AF*: atrial fibrillation, *NYHA* New York Heart association, *IP* immediate post-PTMC, *P* pre-PTMC

In order to identify predictive factors for the occurrence of mitral restenosis, the pre- and immediate post-PTMC characteristics of the patients with and without restenosis were compared with univariable logistic regression. This analysis detected only IMVA < 2 cm^2^ as a significant predictor of restenosis (*p* < 0.001). In the next step the variables with *p* ≤ 0.10 in the univariable analysis were entered into a multivariable logistic regression analysis. Again, IMVA < 2 cm^2^ was the only significant variable (*p* = 0.001). The results of these analyses are shown in Table 5.

**Table 5:** Univariate and multivariate logistic regression analysis results to identify variables predictive of mitral restenosis

Variables	Univariate OR (95% CI)	Univariate *p* value	Multivariate OR (95% CI)	Multivariate *p* Value
Age	1.013 (0.98–1.04)	0.39		
Male Sex	0.972 (0.40–2.30)	0.94		
NYHA functional class > 2	1.144 (0.48–2.69)	0.75		
AF Rhythm	2.008 (0.88–4.53)	0.09	1.977 (0.819–4.774)	0.15
Antibiotic (Penicillin) Prophylaxis	0.732 (0.35–1.50)	0.39		
EF	1.018 (0.936–1.108)	0.67		
Left atrial diameter	1.118 (0.62–1.98)	0.70		
TMPG	1.036 (0.98–1.09)	0.17		
MVA	1.09 (0.54–2.21)	0.78		
MR ≥ grade 1	1.306 (0.63–2.68)	0.46		
Wilkins score > 8	1.845 (0.86–3.93)	0.10	1.886 (0.833–4.268)	0.12
sPAP	0.272 (0.04–1.61)	0.15		
ITMPG	1.159 (0.931–1.444)	0.18		
IMVA ≥ 2cm2	0.231 (0.109–0.491)	< 0.001	0.240 (0.105–0.548)	0.001
IMR: grade 2	2.14 (0.86–5.35)	0.10	1.097 (0.388- 3.101)	0.86

*OR* odds ratio, *CI* confidence interval, *NYHA* New York Heart Association, *AF* atrial fibrillation, *EF* ejection fraction, *TMPG* mean Trans-mitral pressure gradient, *MVA* mitral valve area, *MR* mitral regurgitation, *sPAP* systolic pulmonary arterial pressure, *IMVA* immediate post-PTMC mitral valve area, *IMR* immediate post-PTMC mitral regurgitation, *ITMPG* immediate post-PTMC mean trans-mitral pressure gradient

**Fig. 2:**
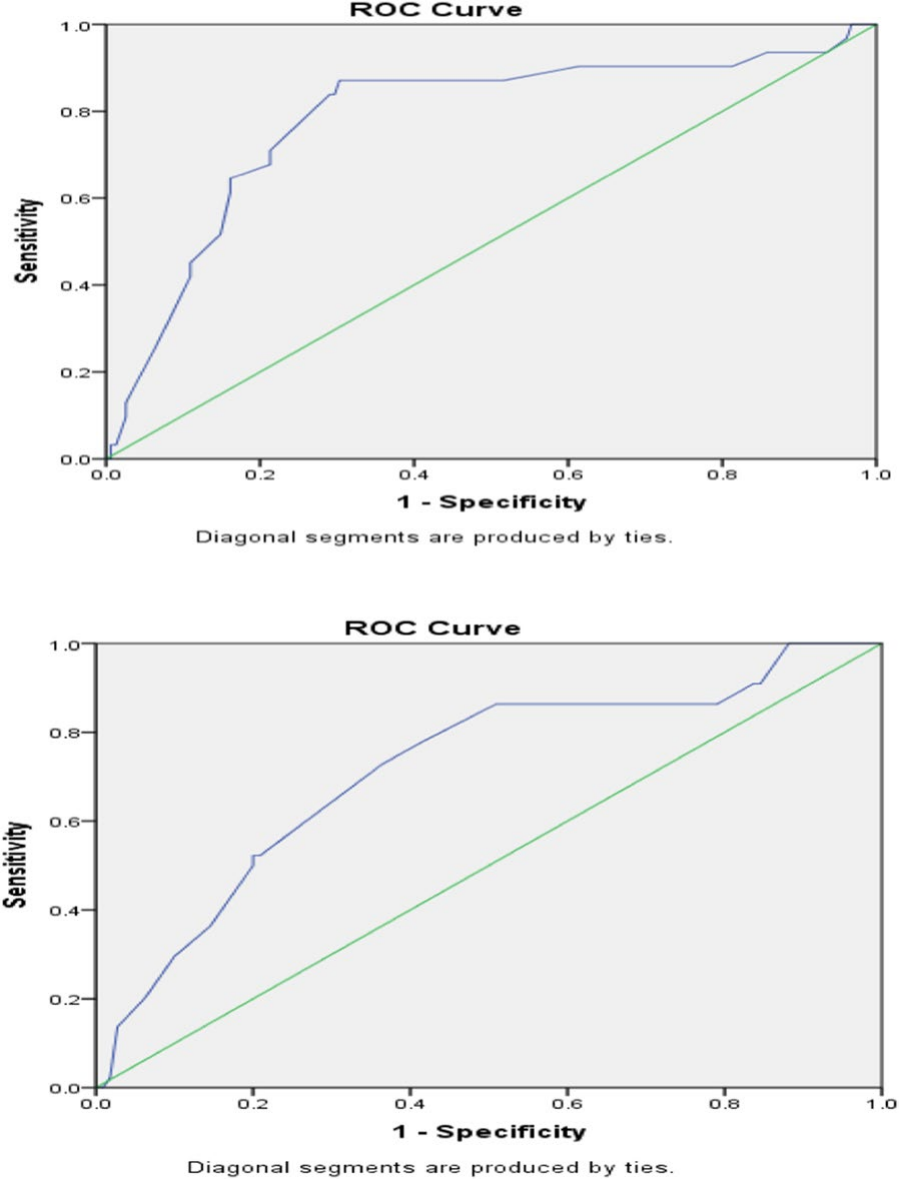
ROC curves to evaluate the optimal IMVA cut-off point for predicting adverse cardiac events [1] and restenosis [2]. The area under curve is 0.784 and 0.717 figure

At last, ROC curves were recruited to obtain optimal IMVA cut-off points for predicting adverse cardiac events (Fig. 2) and also restenosis (Fig. 2); the calculated values were 1.925 cm^2^ and 2.025 cm^2^ respectively.

## Discussion

The main finding of this study is the identification of heterogeneous factors of cardiac rhythm, valvular morphology and primary status of pulmonary arterial pressure as predictors of adverse cardiac events occurring in the long-term follow-up of patients who underwent PTMC for rheumatic MS. Furthermore, low IMVA (< 2 cm^2^) was found to be an independent predictive variable for both adverse cardiac events and restenosis.

To our knowledge this is the second study of the long-term outcomes of PTMC in Iran, and is one of the few such studies in the Middle East region [10, 13, 16]. According to epidemiologic studies worldwide, Iran is no longer an endemic area for rheumatic fever, in contrast to the situation in this country about 4 decades ago [2, 21]. However, we should not underestimate the existing burden of RHD and its major consequences such as MS, while a large group of sufferers are young adults under the age of 50 [1, 2]. This study also reconfirmed that when PTMC is undertaken successfully and without acute complications, there would be a significant decrease in morbidity and mortality among the patients with MS even in a relatively long-term scale, given that 83.3% of our patients had no major adverse cardiac events, 59.6% did not develop restenosis, and 68.2% were still in NYHA functional class ≤ 2 by the end of the follow-up period.

We identified AF rhythm, Wilkins score > 8, l IMVA < 2 cm^2^, and high pre-PTMC pulmonary arterial pressure (reflected as higher sPAP) as independent predictive factors of clinical cardiac events. Several studies have also reported at least one the first three factors to be a prognostic factor of PTMC results in the long-term [11–18, 22].

Aside from thromboembolic events, which can be a direct consequence of AF rhythm, our study is consistent with earlier works reporting that AF rhythm correlates positively with the incidence of cardiac events [13, 14, 16, 22]. Patients with AF in the present study were older and had higher NYHA functional classes and higher Wilkins scores than patients in sinus rhythm, in line with reports by other authors [16, 22]. Bouleti et al. suggested that although AF rhythm per se does not compromise mitral valve function, its presence is an indicator of more advanced stages of underlying rheumatic disease; thus the rate of clinical events will be higher in long-term [14]. A further implication is that impaired hemodynamics and subsequently more severe clinical symptoms in these groups of patients make re-intervention inevitable within a shorter time frame.

Considering Wilkins score, our results are also in line with multiple studies reporting that favorable morphological characteristics before the procedure can play a significant role in the long-term success of PTMC [10, 13, 14, 22]. Although other scoring systems have been introduced, based on estimating variables such as commissural calcium, commissural area or subvalvular characteristics, to date none has been as widely validated or used as Wilkins score [8, 23, 24]. Given that some authors have noted a universal consensus that PTMC will be most effective when valvular and commissural morphology are optimal, the actual impact of PTMC lies in reversing commissural fusion, i.e. the pathognomonic characteristic of rheumatic MS, and in avoiding severe MR – an effect which becomes more likely when the valve is markedly deformed [1, 8, 25, 26].

An unexpected finding in the present study was the detection of higher pre-PTMC sPAP as an independent variable for predicting adverse cardiac events. Although a few studies have reported higher post-PTMC sPAP to be among the predictive factors, none indicated a similarly significant impact for pre-PTMC sPAP [24, 27]. Because of missing data regarding immediate post-procedural sPAP in some cases, we were unable to analyze the impact of this factor separately. However, some studies have shown that patients with high baseline pulmonary arterial pressure had poorer hemodynamic results in intermediate and late follow-up periods [28, 29]. Ozkan et al. suggested that because of the decrease in hemodynamic response to PTMC in patients with severe pulmonary hypertension in the long-term, it would be reasonable to perform PTMC at earlier stages [28]. In light of our parallel clinical finding, we also hypothesize that patients with high pulmonary arterial pressure, despite the documented substantial initial improvement after PTMC, may have surpassed the window of opportunity for this procedure, making it reasonable to opt directly for surgical management in some of these individuals.

The most significant predictive factor in the present study was IMVA, which proved to be clearly influential in predicting both cardiac events and restenosis. This finding is consistent with the results of earlier studies [13, 15, 18, 22], especially that by Song et al. [15]. In contrast with other studies, however, we obtained a slightly higher cut-off value of 2.025 for predicting restenosis and 1.925 for predicting adverse cardiac events [15, 22]. The respective ROC curves to evaluate the diagnostic ability of IMVA are shown in Fig. 2. Song et al. suggested that IMVA is a significant indicator of the efficacy of the procedure, and at least for some more recent target groups of PTMC such as the patients with higher pre-PTMC MVA, a higher cut-off for success than the conventional ≥ 1.5 cm^2^ value should be considered [15]. Tomai et al. also noted that better event-free outcomes in cases with higher IMVA reflect higher procedural quality in this group as well as a less advanced stage of rheumatic MS [22]. We also suggest, based on the present findings, that patients who have less than optimal IMVA results should be followed more closely during follow-up, as they are supposedly more prone to restenosis and more likely to be candidates for re-intervention.

To the best of our knowledge, the present study provides the first analysis of the possible impact of continuing secondary antibiotic prophylaxis on the mid-term outcome in patients who underwent PTMC. Because there was no valid system to monitor patients’ adherence to secondary penicillin prophylaxis at the participating centers, we assumed that patients who did not complete their post-intervention antibiotic regimen might have had a worse clinical outcome. This hypothesis was based on the fact that the repeated occurrence of subclinical rheumatic attacks can affect the post-intervention course of MS. However, this impact was not statistically significant in our results for the incidence of cardiac events or restenosis. In addition, we noted that except for one patient, all those who continued their antibiotic prophylaxis regimen were younger than 50 years of age. This was a logical finding given that based on guidelines for non-endemic area, we recommend that patients continue prophylaxis to around the age of 40. However, our analysis of the results in patients younger than 50 again showed that this impact was statistically nonsignificant. Nevertheless, we suggest that further research on this topic is needed, as only limited number of patients, in other words less than one third of all cases in this study, continued proper antibiotic prophylaxis regimen. Furthermore, regardless of whether continuing the secondary antibiotic prophylaxis or not, the majority of cases in this study suffered from neither adverse cardiac events nor restenosis. Additionally, few valid recommendations have been produced for non-endemic countries, particularly about the duration of continuing antibiotic prophylaxis after PTMC [30]. It is also noteworthy to mention that the points discussed here should not be confused with the proven effect of secondary antibiotic prophylaxis following acute rheumatic fever (ARF) to prevent recurrences of ARF and progression of primary RHD in young patients [1, 30], as all the patients included in this study were proven cases of advanced rheumatic MS at the time of PTMC.

Other factors such as age or NYHA functional class have also been reported in similar studies to be decisive variables in predicting the late outcome of PTMC [14, 15, 18]; however, our findings were inconclusive regarding the impact of these factors, probably because of the smaller size of our study population in comparison to other studies. In addition, all participating hospitals in the present study were referral centers, and as a result more than 75% of the patients included here were in NYHA functional class > 2 at the time of the PTMC. This may have had an impact on the present results.

Additionally, based on our results, ITMPG was not a significant predictor for both adverse cardiac events and mitral restenosis. This is in agreement with the findings of several previous studies [15, 18, 22]. However, there have been reports, in which ITMPG has been singled out as a main predictor of late events, although they had implemented different study designs and endpoints [14, 24]. As Bouleti et al. have reported, ITMPG was the main predictor of the long-term events in older patients, especially after the age of 70 [14]. Nunes et al. have also indicated that they had investigated a heterogeneous group of patients in regard of age and valvular morphology [24]. Therefore, we assume that the different age structure of our study population, in which only 7 patients were of the age of 70 or older have had an impact on our findings in this regard.

It is also noticeable, that in comparison to other similar studies, the rate of left ventricular dysfunction (EF < 50%) at the time of PTMC procedure was lower among our patients (9.13%). We consider the implemented criteria, such as excluding the patients with significant rheumatic involvement of aortic valve or the ones with high grade MR, and again the relatively younger mean age of our case as possible explanations for this difference.

### Limitations

Our study had certain limitations. To obtain an acceptable number of patients, we had to collect data from multiple centers. However, by applying specific criteria and including only cases with immediate success and an initial event-free interval of 6 months, we tried to decrease the operator bias. In contrast, the protocols for echocardiographic imaging and the PTMC technique were completely the same at all centers.

The retrospective nature of this study is an added limitation. We lost a considerable proportion of our patients (15.4%) during follow-up. Additionally, the exact timing of the initiation of restenosis was unknown in some cases, thus we had to apply logistic regression to analyze the results for this factor. Furthermore, the available information about the cause of death in 2 of our deceased patients was limited and mostly based on interviews with first-degree relatives. However, we were able to obtain and recheck the relevant documentation in 2 other cases.

## Conclusion

After a relatively long period of time following PTMC, more than 80% of the patients remained event-free and more than 66% had an acceptable functional status based on NYHA class. The main independent predictors of adverse events were AF rhythm, initial Wilkins score > 8, high initial sPAP, and IMVA < 2 cm^2^. Low IMVA was also the only variable predicting the incidence of restenosis. Although the effect of continuing secondary penicillin prophylaxis on the long-term outcome of PTMC was insignificant in this study, further research on this topic is recommended.

## Supplementary Information

**Additional file 1**. Strobe checklist of patient enrollement.

## Data Availability

Free internet sharing of data are available and we do not use any personal data of our patient in this article.

## Abbreviations

PTMC: Percutaneous transvenous mitral commissurotmy
MVA: Mitral valve area
AF: Atrial fibrillation
HR: Hazard ratio
MS: Mitral stenosis
sPAP: Systolic pulmonary artery pressure
IMVA: Immediate post-PTMC mitral valve area
OR: Odds ratio
ARF: Acute rheumatic fever

## Declarations

### Impact on practice

This is a rare report of late post PTMC result up to 12 years, which showed that AF rhythm, post PTMC MVA, Wilkins score and systolic pulmonary pressure were only predictors of long-term adverse cardiac events. Post PTMC MVA was also the predictor of MS restenosis. It seems that secondary antibiotic prophylaxis may play no role in prevention of restenosis in non-endemic areas.
